# Pleomorphic liposarcoma of the foot: a case report

**DOI:** 10.1186/1746-1596-3-15

**Published:** 2008-04-16

**Authors:** Luka Brčić, Antonija Jakovčević, Lovorka Batelja Vuletić, Dubravko Orlić, Sven Seiwerth

**Affiliations:** 1 Institute of Pathology, Medical School, University of Zagreb, Šalata 10, Zagreb, Croatia; 2 Department of Orthopedics, Medical School, University of Zagreb, Šalata 6, Zagreb, Croatia

## Abstract

**Background:**

Liposarcomas are among the most common sarcomas of adult life. Pleomorphic liposarcoma, characterized by pleomorphic lipoblasts, is the rarest subtype. To our knowledge only three cases of pleomorphic liposarcoma of the foot or ankle have been reported so far.

**Case presentation:**

A 71-year-old female presented with a large growing mass on the dorsum of her right foot. Computed tomography showed invasive tumorous mass. Excision biopsy revealed the mass to be a pleomorphic liposarcoma, and below the knee amputation was performed.

**Conclusion:**

Although the incidence of pleomorphic liposarcoma in the foot is very low, it is essential to perform thorough histological analysis of all soft tissue masses, regardless of their benign appearance, because only prompt radical surgery can result in a good prognosis for the patient.

## Background

Liposarcomas are malignant tumors and among the most common soft tissue sarcomas of adult life [1]. They can be devided according to clinicopathologic and cytogenetic characteristics into three distinct subtypes: well-differentiated, myxoid/round cell and pleomorphic liposarcoma [1,2]. Pleomorphic liposarcomas are the rarest, and are characterized by the presence of pleomorphic lipoblasts. According to recent studies [3-5] pleomorphic liposarcomas occur most often in lower and upper limbs, and only three have been found in foot and ankle, one of them occurring in a burn scar [6].

We present a case of a pleomorphic liposarcoma of the foot, stressing necessity of a proper histological analysis, followed by radical surgery leading supposedly to a better prognosis for the patient.

## Case presentation

**Figure 1:**
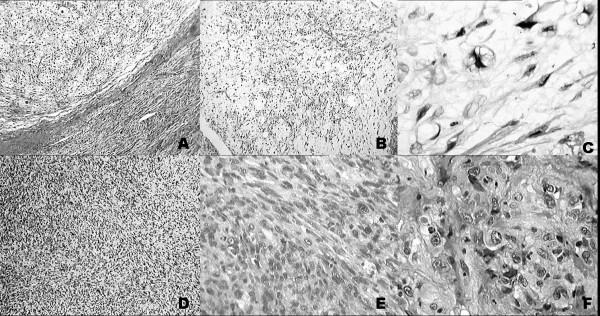
**Histological presentation**. Histological presentation of lipogenic and nonlipogenic areas (A), with pleomorphic lipoblasts (B, C) in lipogenic areas. In nonlipogenic areas pleomorphic spindle cells were interspersed with dense collagen (D, E), while some showed rhabdoid or epitheloid features (F). (hemalaun-eosyn staining; A, B, D-objective ×2; C, F-objective ×25; E-objective ×6.3)

A 71-year-old female of normal weight presented with a large mass on the dorsum of her right foot which has been gradually increasing in size over 4 months prior to admission to the hospital. Two years before she had a soft tissue injury at the same location, but without any complication and sequels. Otherwise she was without serious illness. Her family history revealed no lipomatous disorders. On physical examination a firm, palpable tumor mass, 6–7 cm in diameter, poorly-defined from surrounding tissue and attached to underlying structures and skin, was detected. The mass was painless, unless examined by deep palpation. Skin above it was of normal color and structure. Laboratory findings, including blood count, were completely normal. On plain x-rays only an ill defined mass on the dorsum of the foot, with no clear correlation to the bone, was observed. Computed tomography revealed invasive tumorous mass. Scintigraphy with Tc99 was positive. Magnetic resonance imaging showed expansive infiltrative process of the dorsum of the foot with penetration between metatarsal bones to the plantar side, and partial destruction of the fourth metatarsal bone. Histologically, in biopsy sample, areas with lipogenic characteristics were surrounded by nonlipogenic areas (Figure 1A). In lipogenic areas lipoblasts of different size and shape with variably expressed pleomorphism and number of nuclei were found (Figure 1B,C). The number of lipoblasts also varied considerably from field to field as was the intensity of mixoid change so that some areas had a myxofibrosarcoma-like appearance, a pattern dominating the first biopsy (Figure 1). In nonlipogenic areas sheets of pleomorphic spindle cells with multinucleated giant cells (complying to the pattern of so called malignant fibrous histiocytoma, MFH) were interspersed with more pleomorphic parts or dense collagen (Figure 1D,E). Occasionally some cells showed rhabdoid or epitheloid features (Figure 1F). No significant necrosis was present. A panel of antibodies was studied immunohistochemically: vimentin, S-100, desmin, CD34, CD68, cytokeratin, epithelial membrane antigen (EMA), myoglobin, myogenin and smooth muscle actin (SMA) (all antibodies from DAKO; Copenhagen, Denmark). In tumor cells S-100 was occasionally positive with better expression in lipogenic than in non-lipogenic areas. Positivity for CD68 and myogenin were observed rarely. CD 34 and SMA were positive only in vessel walls, and negative in tumor, while other antibodies yielded no specific staining. Proliferation rate measured with Ki-67 was 40% measured in hot spot areas.

After histological diagnosis below the knee amputation was indicated. No further therapy was indicated, and patient is still alive, 6.5 years after the diagnosis was made.

## Discussion

Pleomorphic liposarcoma is a rare neoplasm, and until know there have been only 3 reported cases of pleomorphic liposarcoma in the foot and ankle. It is known that this neoplasm is usually aggressive, occurring in adulthood, and usually in the limbs. Pleomorphic liposarcoma can occur in mediastinum, liver, orbit, paratesticular region, and also as a purely dermal tumor [3,4,7-11] and are presented as single case studies or in review articles. It usually presents as a painless, deep-seated tumorous mass [3]. It can easily be misdiagnosed, because of the great variety of histological presentations. Histological hallmark of pleomorphic liposarcoma are pleomorphic lipoblast, which can form less than 10% of the tumor. Immunohistochemistry has a limited value in diagnostic procedures. According to Gebhard et al. S-100 protein immunoreactivity can be observed in up to 48% of lipogenic areas, while SMA was positive in 49% of nonlipogenic areas of pleomorphic liposarcoma [3]. They differentiate four morphological subtypes of pleomorphic liposarcoma, based on the appearance of nonlipogenic parts of the tumor: malignant fibrous histiocytoma-like, epithelioid, round cell and spindle cell liposarcoma-like. This classification is in contrast with the view of Enzinger and Weis recognizing only 2 subtypes (MFH-like and with round and pleomorphic cells) [2]. Some dedifferentiated and pleomorphic liposarcomas have been reported to be associated with leukocytosis. This feature, usually expressed in MFH, led to the concept of possible common lineage for MFH, dedifferentiated and pleomorphic liposarcoma [12]. Differential diagnosis includes dedifferentiated liposarcoma, myxofibrosarcoma, leiomyosarcoma, rhabdomyosarcoma, carcinoma, and melanoma, which may be difficult to distinguish especially in small biopsies. This is especially true for dedifferentiated liposarcoma where quality of sampling represents the key parameter in avoiding underdiagnosis. Only adequate sampling will identify both well/and poorly differentiated areas of a given tumor. The distinction from nonlipogenic tumors is based on demonstration of multivacuolated lipoblasts and occasional positivity for S-100 protein in the spindle or poorly differentiated areas. The most complicated task is the differential diagnosis of tumors with highly pleomorphic (MFH-like) features. The differential diagnosis in the groups with round or pleomorphic cells, including epitheloid appearance, largely depends on specific immunohistochemistry for the given tumors. In distinction from poorly differentiated epithelial neoplasms the positivity of occasional cells for epithelial markers should be kept in mind [13]. In all cases inadequate sampling represents one of the basic problems.

Molecular studies so far yielded little useful results: in one third of the patients MDM2 amplification was detected, and TP53 alterations were present in four out of nine patients (all without MDM2 amplification) [14].

We presented a case of a 71-year old female, with tumorous mass on the dorsum of her right foot, which, according to pathohistological analysis, turned out to be a pleomorphic liposarcoma, showing areas of different histology. Our case demonstrates the importance of pathohistological analysis of all soft tissue tumorous masses in the foot/ankle region, which may look like common benign soft tissue lesions, but if not recognized can become life-threatening.

## Conclusion

It is important to bear in mind that the differential diagnosis of pleomorphic lyposarcoma is fairly broad and that thorough sampling and searching for lipoblasts may be rewarding in tumors firstly presenting histologically as one of the mimics (e.g. MFH, myxofibrosarcoma or even metastatic carcinoma or melanoma).

## Competing interests

The authors declare that they have no competing interests.

## Authors' contributions

LB is primarily responsible for drafting, literature search and submission of the manuscript. AJ and LBV vere involved in literature search and preparing the material, DO suplyed relevant clinical information about the patient and was involved in manuscript revision. SS outlined the general concept of the manuscript, has been involved in drafting and revising it critically. All authors have read and approved the final manuscript.
